# Assessing Health System Responsiveness: A Household Survey in 17th District of Tehran

**Published:** 2011-05-01

**Authors:** A Rashidian, Z Kavosi, R Majdzadeh, A Pourreza, F Pourmalek, M Arab, K Mohammad

**Affiliations:** 1 Department of Health Management and Economics, School of Public Health, Tehran University of Medical Sciences, Tehran, Iran; 2 Knowledge Utilization Research Center, Tehran University of Medical Sciences, Tehran, Iran; 3 Director, National Institute of Health Research, Tehran University of Medical Sciences, Tehran, Iran; 4 Department of Management and Medical Informatics, Shiraz University of Medical Sciences, Shiraz, Iran; 5 Department of Epidemiology and Biostatistics, School of Public Health, Tehran University of Medical Sciences, Tehran, Iran; 6 Institute for Health Metrics and Evaluation, University of Washington, Seattle, USA

**Keywords:** Responsiveness, Outpatient services, Inpatient services, WHS, Iran

## Abstract

**Background:**

Responsiveness is an indicator by WHO to evaluate the performance of health systems on nonmedical expectations of consumers. This study measures the health system responsiveness and the factors affecting responsiveness in Iran health system.

**Methods:**

World Health Survey (WHS) questionnaire was used to collect data on a two-stage cluster sampling in 17th District of Tehran in 2003. Of a sample of 773, 677 and 299 individuals who respectively had outpatient or inpatient services utilization responded to the responsiveness module of WHS questionnaire.

**Result:**

More than 90% of respondents believed that responsiveness issues were very important. Performance of outpatient services was better than hospital services in terms of responsiveness. "Prompt attention" and "quality of basic amenities" received low score for outpatient services. Service user variables had no significant effect on responsiveness, while type of centers was significantly related to responsiveness. Principal component analysis found three factors for both outpatient and inpatient services that explained 62% and 61% of total variances respectfully.

**Conclusion:**

Iran health system should pay more attention to responding non-medical expectations of service users. It sounds that health system interventions are main determinant of responsiveness score compared to demographic or user variables. Training health staff, allocating more resources and reengineering some processes may play a role in improving responsiveness. Responsiveness domains seems to be tailored based on each society's cultural factors.

## Introduction

Health systems are expected to meet their core goals as well as a number of common social goals[1] including respecting patient rights and responding to patients' expectations. These have gained particular eminence over the past few decades.[2][3] For these reasons, World Health Organization developed a framework for health system responsiveness. It was first presented in the World Health Report 2000, where it introduced responsiveness as one of the three basic goals of health systems (the other two goals were improving health outcomes and fair financial contribution to healthcare).[4]

Responsiveness is described as fulfillment of people's non-medical expectations while interacting with health system; including the way individuals are treated and the environment in which they are treated.[4] To date, few published work is available about this subject[5] and the use of instrument in this empirical work is little,[6][7][8] while much work has been done on the measurement of patient satisfaction[9][10][11] and quality of care.[11][12][13][14] Responsiveness is different from patient satisfaction and quality of care as it covers health system as a whole, focuses on non-medical aspects of healthcare and evaluates individuals experiences; in contrast, satisfaction is usually limited to a specific healthcare setting such as hospital and considers both medical and non-medical aspects, and represents a complex mixture of perceived needs, expectations and experience of care.[5] Quality of care is also a broad concept which includes technical, process, structural and outcome aspects. Some of the interpersonal dimensions of quality of care have, therefore, been useful in defining the dimensions of responsiveness, but it is claimed that no single quality of care framework incorporates all the domains considered important to responsiveness.[15]

The WHO framework for responsiveness identified a set of domains for the responsiveness concept based on review of patient satisfaction and quality of care literature.[15] They selected the domains that were comprehensive, amenable to self report, and comparable within and across populations.[15] Eight domains including "autonomy", "prompt attention", "confidentiality", "choice of provider", "dignity", "clarity of communication", "quality of basic amenities" were shared between outpatient and inpatient care, and the "social support" domain was considered relevant to inpatient care only. They were also further classified into two groups of 'respect for human rights' and 'client orientation'.

Similar to many health systems, achieving adequate responsiveness remains a challenge for Iran's health system.[16] In WHR2000, Iran health system ranked 100 in terms of responsiveness which indicated urgent need for special attention to healthcare responsiveness.[4] In this study, we evaluated household views on responsiveness of outpatient and inpatient services to their expectations in 2003, as well as the factors affecting responsiveness. We also assessed whether the WHO proposed domains were applicable for evaluating responsiveness of health system in Iran.

## Material and Methods

A household survey was conducted in 2003. A representative sample of households in the 17th District of Tehran was enrolled. The District (population: 260000; households: 71000)[17] is located in southern Tehran, Capital of Iran and has a relatively low socioeconomic status compared with the rest of Tehran.[18] Two-stage cluster sampling approach was used for the survey. In the first stage, 64 clusters were identified using a systematic sampling frame developed by Iran Statistics Center.[19] Each cluster included up to 18 households. Then from each household, an adult individual who was 18 years or older was randomly selected by Kish Table method after completing the household roster.

The original sample covered 1123 households, of which responsiveness questionnaire was completed for 773 households. Each household was approached for data at most 10 times. If no one was available after 10 contacts, the household was substituted by a neighboring household. Responsiveness module of the World Health Survey (WHS) questionnaire, which is a valid, reliable and comparative instrument developed by WHO, was used to collect data. Responsiveness module contains questions about "health services utilization", "importance of responsiveness domains from people view", and "people's view about responsiveness domain of outpatient and inpatient services which were used".[20]

All individual who had used outpatient health services in the past 12 months or inpatient services in the past 5 years were requested to answer the responsiveness modules. It is of note that the study was approved by the Ethics Committee of the Tehran University of Medical Sciences. The participants signed or marked (if illiterate) the informed consent forms. KMO test (0.91 and 0.89 for outpatient and inpatient services respectively) showed that the sample size was adequate for Principal Component Analysis and Bartlett test indicated that PCA was suitable for our study.

## Results

The number of responses depended on the rate of service utilization in the previous 12 months for outpatient care and 5 years for inpatient services. Our results showed that out of 773 individual, 677 (87.5%) reported outpatient services utilization (including primary care, specialist care, dentistry) within past 12 months and 38.6% reported hospital utilization over the past 5 years. They mostly used governmental services, 58.5% and 89% for outpatient and inpatient care respectively (Table 1).

**Table 1:** Health services utilization based on type pf service in 2003

	**Outpatient services**		**Inpatient services**	
	**No.**	**%**	**No.**	**%**
Governmental	395	58.5	269	89
Private	267	39.3	24	9
charity	15	2.2	6	2
total	677	100	299	100

**Fig. 1:**
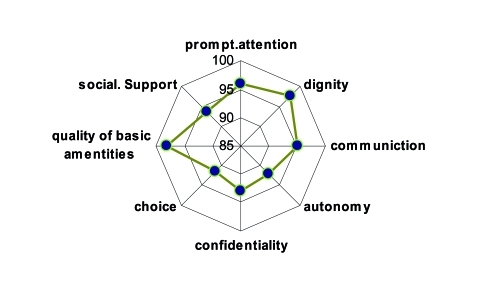
Proportion of people rated responsiveness domains as important or very important

Our finding showed that 6% (42 individuals) could not get care despite that they felt the need to see health care providers. More than half of this group mentioned that they could not afford the cost of the services. Almost 5% of the individuals for whom medicines were prescribed, could not get all the medicines. They reported cost of medicines (40%) and availability of the drugs ('could not find the drugs') (30%) as the main reasons for not obtaining all the prescribed medicines. In answering the question about the involvement of people in decision making about what healthcare to be given and where to receive care, 44% choose "bad" or "very bad" and 26% rated it as "good or very good". Forty nine percent of respondents described their satisfaction with general performance of the health system as "very or fairly satisfied". The majority of the respondents (over 90%) rated all aspects of responsiveness as very important or important: quality of basic amenities (98%), dignity (97.5%), prompt attention (96%) and communication (95%), followed by freedom of choice, and autonomy (92%) (Figure 1).

**Fig. 2:**
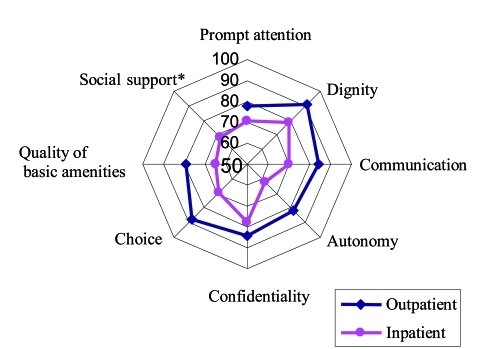
Comparing responsiveness domains of inpatient and outpatient services in 2003. (*Social support is just applicable for inpatient services)

Figure 2 shows the proportion of people reported responsiveness of inpatient services they received as "good or very good" in all domains. The best performing domains were dignity and confidentiality (both at 78%). The worst performing domains were autonomy (62%) and quality of basic amenities (65%). The scores of outpatient service responsiveness domains are demonstrated in Table 2. The best performing domains were dignity and autonomy (90% and 87% respectively). Autonomy and quality of basic amenities (both 78%) received the lowest scores.

**Table 2:** Component matrix for outpatient/inpatient services responsiveness

	**Outpatient (component)**			**Inpatient (component)**		
	**1**	**2**	**3**	**1**	**2**	**3**
**Prompt attention**	-	-	-	-	-	-
traveling time	-	-	0.832	-	-	0.736
time waited	-	-	0.699	-	-	0.743
**dignity**	-	-	-	-	-	-
being greeted and talked to respectfully	0.631	-	-	-	-	0.665
privacy during physical examinations and treatments	0.627	-	-	-	-	0.667
**communication**	-	-	-	-	-	-
clarity of providers explanations	0.813	-	-	0.606	-	-
time to ask questions about health problem/treatment	0.826	-	-	0.574	-	-
**autonomy**	-	-	-	-	-	-
getting information about other types of treatments/tests	0.739	-	-	0.686	-	-
being involved in making decisions about care	0.701	-	-	0.581	-	-
**confidentiality**	-	-	-	-	-	-
talk privately to health care providers	-	-	-	0.737	-	-
confidentiality of personal information	-	0.601	-	0.678	-	-
**choice**	-	-	-	-	-	-
freedom to choose health care provider	-	0.607	-	0.575	-	-
**Quality of basic amenities**	-	-	-	-	-	-
cleanliness of the rooms inside including toilets	-	0.851	-	-	0.734	-
space in the waiting & examination rooms	-	0.820	-	-	0.787	-
**Social support**	-	-	-	-	-	-
The ease of having family & friends visiting	-	-	-	-	0.797	-
Staying in contact with the outside	-	-	-	-	0.782	-

We created a binary variable (those ranking responsiveness as 'good' or 'very good' in one group, others as the other group) and then used Chi-Square test and compared the characteristics of the respondents such as sex, education, ethnicity, marital and socioeconomic status and provider variables including type of center and sex of provider. No significant differences were found in ranking responsiveness domains in terms of sex, education, ethnicity, marital and socioeconomic status for outpatient and inpatient service users. Binary regression analysis confirmed the effect of type of center on responsiveness (i.e. private centers were more responsive). Comparison of responsiveness aspects in governmental, private and charity hospitals showed that private hospitals performed better in all aspects (p-value < 0.001) (Table 3).

**Table 3:** Percentage of people rated responsiveness domains of outpatient services as good or very good based on type of center

	**In/Outpatient**			**Inpatient**		
Type of center	Governmental	Private	Charity	Governmental	Private	Charity
Domains						
Prompt attention	77	80	58	70	89	50
Dignity b	88	92	92	75	84	73
Communication b	81	89	67	70	78	64
Autonomy b	78	87	66	62	62	60
Confidentiality b	81	88	91	78	88	60
Quality of basic amenities b	77	83	83	67	72	28
Choice b	67.5	79	75	62	56	50
Social support	NA^a^	NA	NA	70	72	54

^a^ NA. Not applicable for outpatient services

^b^ p-value < 0.001

Comparison of responsiveness domains based on outpatient center type also showed significant differences, in a way that private centers performed better in all aspects (p-value < 0.001) (Table 3). Almost 10% of respondents perceived some sort of discrimination while receiving inpatient services. No differences in this regard were found between public and private centers. No one reported being discriminated against because of their nationality or ethnicity. On the other hand, lack of money and social class were the main reported reasons for discrimination.

Principal Component Analysis revealed that three main components explain 61% of the variance for inpatient services responsiveness. First component including communication, autonomy, confidentiality and choice explained 21.9% of variances, 2nd component included quality of basic amenities and access to social support and 3rd component included prompt attention and dignity domains.

PCA for outpatient services responsiveness also found three components explaining 62% of the variances. First component included dignity, communication and autonomy domains, 2nd component included quality of basic amenities and social support and the 3rd component included prompt attention domain.

## Discussion

Except for one article published in Persian on responsiveness in children care,[21] this is the first research paper from Iran about health service responsiveness, while several studies on patient satisfaction and quality of care are available in literature.[22][23][24][25] It is also of importance that as WHS is valid, comparable instrument for responsiveness assessment developed by WHO, its use provides a condition to compare the results with the ones of other studies in other countries. On the other hand, results of PCA provide new information for interested researchers of this subject to revise the questionnaire based on it.

This study has several important implications. People used governmental health services more despite its worse rate in comparison to private sector in terms of responsiveness. Studies in South Africa also indicated that people used governmental health services more.[7] As our study population is in a low income district of Tehran, it seems that economic ability of households could be a main factor for using governmental services, especially as the majority of respondents reported that responsiveness aspects were very important to them. This may also demonstrate a geographical access issue, as most private hospitals are located in North, North West and Center of Tehran.

Dignity, quality of basic amenities, prompt attention and communication received higher scores in terms of their importance. This is somehow similar to a study of patient expectation in Iran.[26] A review of patient expectation study also showed items related to quality and communication that received more importance.[26] Hospital responsiveness domains scores were lower on autonomy, quality of basic amenities, prompt attention and social support. Autonomy, namely getting information about type of treatment and involving consumer (patients) in decision making about treatment and care options, seems not have been given sufficient attention from health care providers. Hence, training and orientation of medical and nursing staff are important as autonomy is likely to be beneficial not only in improving the welfare of the individuals in their interactions with the health system but also in the health outcome of care due to better compliance.[5][26] Fairly low scores in prompt attention and quality of basic amenities highlights the point that improvement of this domain is somehow resource-dependent,[27] however reengineering of patient admission process is undoubtedly effective in improving prompt attention. Evans et al. found a positive relationship between prompt attention and per capita income of country, in a way that countries with higher per capita incomes received higher score in prompt attention domain.[28]

Comparison of the scores of responsiveness component of hospital services from this District in Tehran, Capital of Iran and other countries showed that the score in our study was better than South Africa in all aspects except quality of basic amenities and social support,[7] and the scores were worse than Brazil in all aspects except prompt attention,[29] and worse than European countries in all aspects.[30] For European countries, social support was the best performing domain.[30]

In our study, out-patient responsiveness scores were better than South Africa[7] in all domains, better than Brazil in prompt attention, communication and autonomy domains[29] but worse than European countries in communication, autonomy, confidentiality and quality of basic amenities.[30] The relative rankings of domains among outpatient services were the same as other countries with the lowest scores given to prompt attention and the highest to dignity.

Comparison of outpatient and inpatient responsiveness indicated higher scores of outpatient services in all domains. It seems Iran health system should pay more attention to inpatient services and change the criteria of hospital evaluation in order to include responsiveness domains. Clinical governance initiative, recently started in Iran, may play an important role in improving quality of care and responsiveness to patient expectations.[31]

Using 0.4 as factor loading cutoff point in Principal Component Analysis to determine whether or not the domains should be considered a good factor showed that "communication, autonomy, confidentiality and choice" of inpatient services were loaded in one factor rather than being unique factor as proposed by WHO. Quality of basic amenities and access to social support were loaded in one factor; and lastly, prompt attention and dignity were loaded in one factor. For outpatient services responsiveness, the dignity, communication, and autonomy were load in one unique factor. It seems that in our sample of Iranians, talking respectfully, giving enough information, listening to patient and involving him/her in decision making are interrelated and have less difference among the respondents. "Prompt attention" was loaded in one unique factor as proposed by WHO. Similarly, Peltzer (2009) found that communication, autonomy and confidentiality are the main factors.[7] In Taiwan study, five factors including respect, access, confidentiality, basic amenities and social support were extracted.[8] Considering the similarities, comparing results of Principal Component Analysis of this study, South-Africa and Taiwan studies with WHO responsiveness domains highlights the importance of cultural specification of responsiveness aspects. Our study had some limitations. We collected self-reported subjective data rather than objective data. The long recall period of 5 years for inpatient services use, as proposed by the WHO proning to recall biases was the other limitation.

In conclusion, responsiveness domains especially prompt attention and quality of basic amenities of outpatient/inpatient services were the priority area for improvement. As the type of provider was a main contributor to responsiveness and service users, socioeconomic variables had little effect on responsiveness. It sounds that health policy should concentrate on provider performance,[31] training them about responsiveness issues and injecting more resources for improving responsiveness domains. Considering that this low income population used governmental services more on one hand, and gave lower scores to governmental services responsiveness on the other hand, further studies on the equity aspects of service use and responsiveness seem warranted. As responsiveness is considered a health system goal, studies that monitor the performance of health system in responsiveness and the effects of interventions on it will be of importance.
